# Prevalence of Depression and Dementia among Adults with Developmental Disabilities in Manitoba, Canada

**DOI:** 10.1155/2011/319574

**Published:** 2011-08-08

**Authors:** Shahin Shooshtari, Patricia Joan Martens, Charles A. Burchill, Natalia Dik, Saba Naghipur

**Affiliations:** ^1^Departments of Family Social Sciences and Community Health Sciences, University of Manitoba and St. Amant Research Centre, Manitoba, Canada R3T 2N2; ^2^Manitoba Centre for Health Policy, Department of Community Health Sciences, Faculty of Medicine, University of Manitoba, Winnipeg, Manitoba, Canada R3E 3P5; ^3^Faculty of Science, University of Manitoba, Manitoba, Canada R3T 2N2

## Abstract

*Study Objective*. To estimate and compare the prevalence of dementia and depression among adults with and without developmental disabilities (DDs). *Methods*. We linked data from several provincial administrative databases to identify persons with DDs. We matched cases with DD with persons without DD as to sex, age, and place of residence. We estimated the prevalence of dementia and depression and compared the two groups using the *Generalized Estimating Equations* (GEEs) technique. *Results*. The estimated prevalence of depression and dementia among younger adults (20–54) and older adults (50+) with DD was significantly higher than the estimated rates for the matched non-DD group (*Depression*: younger adults: RR = 2.96 (95% CI 2.59–3.39); older adults: RR = 2.65 (95% CI 1.84–3.81)), (*Dementia*: younger adults: RR = 4.01 (95% CI 2.72–5.92); older adults: RR = 4.80 (95% CI 2.48–9.31)). *Conclusion*. Significant disparities exist in mental health between persons with and without DDs.

## 1. Introduction

Persons with developmental disabilities (DDs) are those who “have significantly greater difficulty than most people with intellectual and adaptive functioning and have had such difficulties from a very early age. Adaptive functioning means carrying out everyday activities such as communicating and interacting with others, managing money, doing household activities and attending to personal care” [1]. DD is often used by researchers as a broad term to include a range of neurological diagnoses. In this paper, we report the estimated prevalence of dementia and depression for adults with DDs, intellectual disability (ID), and autism spectrum disorders (ASDs). 

Although prior research shows that life expectancy for persons with DDs, and in particular for those with Down syndrome, has increased significantly over the last few decades [2], significant health disparities remain between persons with and without DDs (e.g., [3, 4]). Of great concern are disparities in mental health; a large proportion of persons with moderate to severe DDs (upwards of 30–60%) reportedly have mental disorders [5]. Extensive information exists on the mental health needs and the prevalence of specific mental illnesses among persons with DD from the United States and several European countries (e.g., [6–15]). However, the literature on the mental health needs of persons with DD in Canada remains very limited [16–25]. Most Canadian research has focused on hospitalizations for mental health or psychiatric disorders [16, 17, 19–21, 23–25] and has centered on the Canadian province of Ontario [19–21, 23–25]. Some of these studies focused on a small number of persons from a selected unit or hospital [22]. 

The most recent Canadian study [16], also based on hospitalization records, but nationally, used data administered by the Canadian Institute of Health Information (CIHI). This study concluded that, overall, 41.5% of all Canadian hospitalizations among persons with DD occurred for psychiatric reasons. Although Canada has an estimated 120,000+ individuals aged 15 or older living with DDs [26], no population-level information exists on the prevalence and epidemiology of mental illnesses among this population living in the community or on their mental health needs. 

People living with a dual diagnosis of DD and mental illness have complex health and social needs, therefore requiring ongoing support for health, housing, education, and employment. Late diagnosis and inadequate support could result in significant economic cost and further complications both for these individuals and for their families and caregivers [27]. We need accurate estimations of the prevalence of mental illnesses in this population, along with other demographics and health-related information, to better identify their medical and social support service needs. To address this lack, we conducted the present study using linked data from several provincial administrative databases in Manitoba to (1) estimate the prevalence of two types of mental illnesses, dementia and depression, among younger (20–54) and older (55+) adults with DD; (2) compare the age-specific prevalence estimates between persons with DD and a matched comparison group without DD. We focused on dementia and depression because prior research yielded high, but inconsistent, prevalence estimates. Moreover, research indicates that these two illnesses are a growing source of functional decline, morbidity, and poor quality of life (e.g., [2, 27]). 

Dementia is a mental disorder characterized by a loss of intellectual abilities of sufficient severity to interfere with social or occupational performance [28]. Although some studies have examined the risk of dementia among people with DD, only a few were population-based studies. Most of these studies—conducted in the USA, UK, Ireland, and Australia—focused on the prevalence of dementia only among subjects with ID. These studies produced inconsistent results. For example, a state-wide survey in New York State found an overall prevalence of 3.1% among individuals with ID aged 40 and older. The prevalence among persons with ID aged 60 and over was estimated at 6.1% [29]. In Ireland [30], Tyrrell and colleagues (2001), using DSM-IV criteria for dementia, estimated age-specific prevalence at 1.4% for those aged 40 and under, 5.7% for those aged 40 to 49, and 30.4% for those aged 50 to 59. A recently published report on dementia in older adults with ID [31] highlights the considerable variation found amongst studies in terms of dementia prevalence. 

Depression, another mental health disorder shown to affect persons with DD at a higher rate than the general population, is a common disorder that presents with depressed mood, loss of interest or pleasure, feelings of guilt or low self-worth, disturbed sleep or appetite, low energy, and poor concentration [32]. National data from the *Canadian Community Health Survey: Mental Health and Well-Being* (CCHS 1.2) estimates the lifetime prevalence of major depressive episodes among Canadians aged 15+ at 12.2% [95% CI, 11.7–12.7%] [33]. Using health administrative data, one report estimated the five-year treatment prevalence of depression for the population of Manitoba, Canada, at 18.2% (18.09–18.29). It reported that the highest prevalence of depression among residents of Manitoba over a five-year time period occurred in adults aged 35 to 54 [34]. 

Although reports of depression prevalence exist for persons with DD, they provide fairly inconsistent results. Moreover, most prior studies focus on the risk of depression among persons with ID only. For example, a study by Deb and colleagues in South Wales, UK (2001) screened a group of 90 randomly selected adults with ID aged 16 to 64 for various psychiatric illnesses, using the Mini Psychiatric Assessment Schedule for Adults with Developmental Disability (Mini PAS-ADD) and using criteria from the International Classification of Diseases-10th revision (ICD-10). Using both criteria, the study estimated the prevalence of depressive disorder at 2.2% [9]. More recently, Bhaumik and colleagues investigated the psychiatric diagnoses of 2711 adults with ID aged 19 and older in Leicestershire and Rutland, UK [14]. All diagnoses were recorded based on clinical assessments using ICD-10 criteria. The study estimated the overall prevalence of depressive syndrome at 4.3%. In contrast to these results, Marston et al. (1997) used a standard checklist comprising 30 symptoms from ICD-10 diagnostic criteria and the Disability Assessment Schedule [8]. The checklist was completed with the clients and caregivers for 82 persons with ID aged 15 to 58 in the West Midlands, England. Marston estimated the prevalence of depressive syndrome at 43.9%. As the reviewed literature makes evident, the majority of prior studies focused on the risk of depression only among persons with ID and reported variable estimates. The substantial variations in reported rates could be due to several factors, including the specific type of ID studied, study population characteristics (e.g., age and sex), depression criteria, and methods of measurement. 

Given the existing inconsistencies in the reported estimates and the lack of population-based information on the prevalence of depression and dementia among persons with DD in Manitoba, we conducted the present study using population-based linked administrative data from several sources to (1) estimate the prevalence of depression and dementia among younger (20 to 54) and older (55+) adults with DD living in Manitoba and (2) compare their estimated rates to those of a matched comparison group without DD. 

## 2. Methods

### 2.1. Region and Population

Manitoba has a population of 1.17 million, making it the 5th largest of Canada's provinces and territories. Children under the age of 15 make up 19.6% of Manitoba's population, whereas seniors aged 65 and over represent 14.1% [35]. Manitoba has a relatively large aboriginal population (12.7%), which has a much higher reported rate of disability (e.g., [36]). The target population of our study comprised Manitoba residents of all ages. We used the December 31, 2000, population count from the Manitoba population Registry for prevalence estimation. 

### 2.2. Data Sources

This study analyzed five fiscal years of data (from April 1, 2000, to March 31, 2005) from multiple administrative databases contained in the Manitoba Population Health Research Data Repository (hereafter, the Repository). The Manitoba Centre for Health Policy (MCHP) of the University of Manitoba maintains the Repository, which contains a comprehensive collection of anonymized but linkable health and nonhealth administrative databases covering all Manitoba residents. Researchers have used the Repository extensively for population-based research and the quality of the Repository's data has been evaluated as high based on the completeness of the data sets and the accuracy of the information recorded [37–41]. The following section briefly describes the administrative data sets used in the present study. 

#### 2.2.1. Population-Based Registry

The registry contains demographic information, such as age, sex, and location of residence, for all Manitoba residents (including those living in First Nations communities) registered with the provincial department of health, Manitoba Health (MH), to receive publicly funded universal health care. Thus, it provides an accurate number of the population of Manitoba to serve as the denominator for the calculation of age-specific rates. We used information on age, sex, and place of residence for matching purposes. 

#### 2.2.2. Administrative Health Databases

The provincial government originally developed the administrative health databases in 1970 to administer the universal medical insurance plan. The Repository at MCHP contains anonymized records of these databases for virtually all contacts with the provincial health care system, including physicians, hospitals, personal care (nursing) homes, and home care, as well as pharmaceutical prescriptions. All individuals registered in the provincial health care program are assigned a nine-digit Personal Health Identification Number (PHIN). MCHP uses an encrypted, scrambled version of the PHIN as the consistent nonidentifying research number, which permits researchers to link data across data files and track individuals over time while ensuring confidentiality. This study used data from three health administrative databases. First, we used the *Hospital Abstracts database*, which contains information taken from medical charts created when patients are discharged from hospital. It includes demographic as well as clinical information relating to inpatient and outpatient services received [42]. The clinical information in the database, which uses the ICD-9-CM (the International Classification of Diseases— 9th Revision—Clinical Modification), codes up to 16 diagnoses and 12 surgical procedures [43]. As of April 1, 2004, the ICD–10-CA and CCI, the 10th revision of the ICD coding system, codes up to 25 diagnoses and 20 procedures. This study searched all hospital discharge codes when looking for a diagnosis.

Second, we used the *Physician Claims database*, which contains information from records of patient contacts with physicians. This database has the primary purpose of financially reimbursing health care providers for services provided. The clinical information on physician claims includes only one diagnosis coded using ICD-9-CM [43]. Third, we used the *drug database, *which contains prescription drug claims from the Drug Programs Information Network (DPIN), an online point-of-sale prescription drug database for all community-based pharmaceuticals dispensed to Manitoba residents since 1994.

### 2.3. Developmental Disability Case Definition

Using data from the Repository, we identified all persons living with a DD (i.e., cases) in Manitoba during the five-year study period from April 1, 2000, to March 31, 2005. These cases included those meeting at least one of the following three criteria: (1) received income assistance for reasons of ID, from Manitoba Department of Family Services and Consumer Affairs; (2) received special education funding from Manitoba Department of Education for reasons of multiple handicaps, usually defined as ID plus one or more physical disabilities; (3) had at least one ICD diagnostic code for ID and/or ASDs in either the physician claims or the hospital abstracts database. The ICD diagnostic codes used in this study to identify cases with ID included 317 (mild mental retardation (MR)), 318 (moderate, severe, and profound MR), 319 (unspecified MR), 760 (fetal alcohol spectrum disorders (FASDs)), and 758 and 759 (chromosomal and congenital anomalies associated with MR, including Down's syndrome, Patau's and Edward syndromes, Fragile X syndrome, and Prader Willi syndrome). The ICD–10–CD that we used included F700–F701; F708–F711; F718–F721; F728–F731; F738–F739; F780–F781; F788–F791; F798–F799; F840–F841; F843–F845; F848–F849; P043; Q860–Q862; Q868; Q870–Q873; Q875; Q878; Q898; Q900–Q902; Q909–Q917; Q930–Q939; Q992. 

### 2.4. Study Period

We based all analyses presented in this paper on five fiscal years of data: from April 1, 2000, to March 31, 2005. 

### 2.5. Study Population

The total number of individuals of all ages who met at least one of our DD criteria was 7,362. Of these, 7,135 persons had at least 365 days of coverage. Of these, we excluded those residing in personal care homes (PCHs), that is, a nursing home, or having a Public Trustee postal code. We made this decision for several reasons. First, we suspected that cases with DDs in PCHs and those listed with a Public Trustee postal code would have higher rates of comorbidities, including mental illnesses, making their health profiles quite different from those living independently in their communities. Second, compared to the general population, those in PCHs or in other residential facilities have a different level of access to health and social services; therefore, including them might introduce some bias to our results. Third, we excluded such cases because, in some cases, it was difficult to match them according to their place of residence. We also excluded ten people from the study group because they had no assigned Income Quintiles (IQ) (An income quintile divides the population into five income groups (from lowest to highest income) with (approximately) 20% of the population in each group. We derived the income quintiles with data from the public-use census file 2001 developed by Statistics Canada. We linked the census file to the Manitoba population from the Manitoba Population Registry via the corresponding postal code conversion files) information attached to them in the database. This left us with a total of 6,054 individuals who lived for at least one full year between 2000 and 2005 in the community and who met our DD criteria. Of these 6,054, we matched a total of 6,027 by sex, age (year of birth), and three-digit postal code, using a ratio of 1 : 2. We matched for age, since persons with DDs are found to be a much younger population than the non-DD population. In addition, research has shown that the prevalence of some mental illness conditions—for example, dementia—increases with age [2]. We matched for sex, as research shows that a higher proportion of persons with DD are males than females [44]. In addition, the conditions of interest, depression and dementia, are more commonly found among females [34, 45]. We also matched the two study groups based on place of residence, since research shows that this affects individuals' access to health services (e.g., [46, 47]). 

We matched a total of 27 people on the basis of the same characteristics, but using a ratio of 1 : 1. We could not match the remaining six individuals, so we excluded them from the analysis. Thus, our DD population comprised a total of 6,048 persons with DD living in the community. We based the analyses presented in this paper on data for those aged 20+ with DD (*n* = 1,619) and their matched comparison group (*n* = 3,231). 

### 2.6. Study Measures

#### 2.6.1. Prevalence of Dementia

We determined a history of dementia using the ICD diagnostic codes from the medical records, including both the Physician Claims and Hospital Abstracts databases, using five years of data—2000/2001 to 2004/2005. We considered the presence of any of the ICD-9-CM codes of 290–292 (organic psychotic conditions), 294 (other organic psychotic conditions), 331 (cerebral degenerations), or 797 (senility) in either database an indication of dementia. ICD–10–CA codes included F00, F01, F02, F03, F04, F05.1, F06.5, F06.6, F06.8, F06.9, F09, F10–F19, G30, G31.0, G31.1, G31.9, G32.8, G91, G93.7, G94, and R54. 

#### 2.6.2. Prevalence of Depression

We determined a history of depression via the ICD diagnostic codes from the medical records, as well as the prescribed medication database, using the following criteria:

- at least one hospitalization with a diagnosis of depressive disorder, affective psychosis, neurotic depression, or adjustment reaction [ICD–9–CM codes 296.2–296.8, 300.4, 309, 311; ICD–10–CA codes F31, F32, F33, F341, F38.0, F38.1, F41.2, F43.1, F43.2, F43.8, F53.0, F93.0];
- at least one physician visit with a diagnosis for depressive disorder, affective psychosis, or adjustment reaction [ICD–9–CM codes 296, 309, or 311];
- at least one hospitalization with a diagnosis for anxiety disorders [ICD–9–CM code 300; ICD–10–CA codes F32.0, F34.1, F40, F41, F42, F44, F45.0, F451, F452, F48, F68.0, F99] and one or more prescriptions for an antidepressant or mood stabilizer;
- at least one physician visit with a diagnosis for anxiety disorders [ICD–9–CM code 300] and one or more prescriptions for an antidepressant or mood stabilizer.

This operational definition of depression has been validated and follows prior health research using MCHP health administrative data sets [47, 48].

We calculated prevalence estimates in two different ways: (1) as the number of people with the chronic condition of interest per 100 population and (2) as the number of people with the chronic condition of interest per 1,000 person-years (PYs), to take into consideration years of coverage over the five years of the study period. Note that administrative data do not directly identify people who had a particular disease of interest; rather, they indicate who used health services for that particular disease or illness. 

### 2.7. Data Analyses

As mentioned earlier, we calculated the prevalence of dementia and depression in two different ways. For descriptive analyses, we reported prevalence as the proportion of the population (%) with dementia and depression. We used Generalized Estimating Equations (GEE; SAS PROC GENMOD, with repeated subject) to test if the rates of dementia and depression (calculated as the number of people with the conditions of interest, that is, depression and dementia, per 1,000 PYs) were statistically higher or lower than the estimated rates for the matched comparison group. The GEE is a method of analyzing correlated data, where the exponent of the estimate indicates the risk ratio (RR) [49]. We used the RR and 95% confidence intervals to determine the statistical significance of the observed differences in prevalence estimates. We performed programming and data analyses using SAS software, version 9.1. 

### 2.8. Ethics

The University of Manitoba Health Research Ethics Board, the Health Information Privacy Committee (HIPC) of Manitoba Health, Department of Family Services and Consumer Affairs and the Manitoba Department of Education approved this research. 

## 3. Results

Between April 2000 and March 2005, a total of 6,048 persons of all ages lived with DD in Manitoba—64.15% males and 35.85% females. As shown in Table 1, slightly higher than 73% of the population with DD were children under the age of 20; 1,619 were adults aged 20+. Of these, 1,401 (or 86.5%) were between the ages of 20 and 54, and 218 (or 13.5%) were 55 years of age or older (Table 1). Of the 218 individuals with DD aged 55+, 101 were males and 117 were females.

As shown in Table 2, among persons with DD aged between 20 and 54 (*N* = 1,401), a total of 73 had a diagnosis of dementia during the five-year study period. This translates into a prevalence of 5.21% (95% CI 4.05–6.37). DD cases aged 55+ had almost triple the prevalence at 13.76% (95% CI 9.19–18.33). Results of statistical testing using the GEE technique showed that a significantly higher estimated prevalence of dementia among both younger adults and older adults with DD than for the matched comparison groups without DD. As summarized in Table 4, we estimated the RR for dementia for the younger adult group at 4.01 (95% CI 2.72–5.92); for the older age group, we estimated the RR at 4.80 (95% CI 2.48–9.31). 

As shown in Table 3, 595 (42.47%) of the DD study cohort aged 20 to 54 had a diagnosis of depression during the five-year study period. Among the older population with DD, 84 (38.53%) had a diagnosis of depression. Statistical testing using the GEE technique showed that the estimated prevalence among both younger adults and older adults with DD was significantly higher than the estimates for the matched comparison groups without DD. As Table 4 shows, we estimated the RR for depression at 2.96 (95% CI 2.59–3.39) for the younger adult group and at 2.65 (95% CI 1.84–3.81) for the older age group (55+).

## 4. Discussion

The present study extended previous research on the prevalence of mental illnesses among persons with DD by designing a population-based comparative study using the linked data from several administrative databases, covering the entire population of a Canadian province. This study was the first attempt in Manitoba to use a record linkage technique to estimate the prevalence of specific mental illnesses, including depression and dementia, at the population level for persons with DDs. A strength of our study was the use of unique identifiers to link records. This helped us to identify a large number of persons having DDs and the health conditions of interest without duplication. Our methodology, which linked multiple sources of data, improved our ability to identify DD cases living with diagnosed depression or dementia anywhere in Manitoba over the five-year study period. 

Overall, consistent with previous research, our findings showed a significantly higher prevalence of dementia and depression in the DD population than in the matched non-DD population. More specifically, we found a four times higher risk of dementia among younger adults with DD than for the matched non-DD group (RR = 4.01; 95% CI (2.72, 5.92)); the risk was almost five times higher for the older age group [RR = 4.8; 95% CI (2.48, 9.31)]. Our estimate of the prevalence of dementia in the younger age group, 5.21%, aligns with estimates from previous population-based studies conducted in the USA, Ireland, and the Netherlands (e.g., [29, 50]). Our estimate of the prevalence of dementia in the older age group, 13.76%, is lower than some previously reported estimates (e.g., 30.4% reported by Tyrrell and colleagues) [23], but it aligns with the 13.1% estimate for persons with DD aged 60+ reported by Strydom and colleagues [51]. 

We found a similar pattern for depression. The risk of depression among younger adults with DD was almost three times higher than for the matched non-DD population (RR = 2.96; 95% CI (2.59, 3.39)). We found a 2.6 times higher risk of depression among older adults with DD than for the matched non-DD population (RR = 2.65; 95% CI (1.84, 3.81)). Our estimate of the risk of depression among the non-DD population, in both the younger (19.97%) and older age groups (19.18%) in our study, was very similar to the reported five-year estimate of depression among the general population of Manitoba aged 10+ (18.2%) [34]. 

We estimated the prevalence of depression for the younger DD population at 42.5%, which agrees with the reported estimate of 43.9% in a study conducted in the West Midlands in England [8]. It is, however, much higher than the reported estimates of 2.2% [9], 4.3% [14], 8% [11], or 11.1% [7] previously reported. The huge variations found in the prevalence estimates might arise from a number of factors, including variations in the studied populations. Our study used administrative data to identify DD cases and included those with IDs and/or ASDs. Some previous studies focused on the risk of depression among persons with ID only [7–9], autism only [10], or a specific type of ID only (e.g., Down syndrome) [12]. Other possible contributing factors include the data source and the type of information gathered to identify cases with depression. This study used population-based administrative data to identify cases with depression. More specifically, we used clinical diagnoses or information about prescribed medications to identify those with indications of depression. This method of identifying cases differs from the methods used in prior studies. Some studies used standardized checklists—for example, the Psychopathology in Autism Checklist [15]—or involved interviews with clients and their care providers (e.g., [8]). In addition, some studies only considered cases with severe depression (e.g., [14]). Our study did not distinguish among different types of depression and included all diagnosed cases. 

Note that, overall, our reported rates of depression and dementia seem high; this could be due to our reliance on diagnostic codes rather than the definitive diagnoses made by trained specialists for depression or dementia among this particular population. Thus, we may well have included many mild forms of depression not normally included in severe and major depression criteria, as well as cases of misdiagnosed dementia. Over the last few decades, many scholars and researchers have noted and discussed the complexity of diagnosis and taxonomy of psychopathological conditions in persons with DDs. For example, Verhoeven and Tuinier (1997) emphasized the atypical presentation of psychopathological conditions in persons with mental retardation (MR). They described atypical psychopathological presentations, for instance—such as self-injurious behaviour and other challenging behaviours—as symptoms of depressive disorder [52]. In addition to atypical presentations of psychopathological conditions by persons with MR, evidence from factor analytic studies indicates that specific psychopathological conditions or symptoms, not classified elsewhere, exist among persons with MR [53, 54]. Therefore, others have questioned the utility of routine diagnostic criteria for major psychiatric diagnosis among persons with DD, and in particular for those with MR. In this study, we used administrative data sources and mainly clinical diagnoses to estimate prevalence of depression and dementia. Thus, it is important to note that our prevalence estimates are reflective of “administrative prevalence” rather than the true prevalence of conditions at the population level. The true prevalence of the conditions of interest can only be obtained by screening population using standard diagnostic criteria and clinical assessments or by means of well-designed epidemiological surveys. 

Our study has several other limitations relevant to any interpretation of the results. First, we used administrative data to identify DD cases. Although we used several administrative databases, we very likely failed to identify all DD cases living in Manitoba, since some people with DD may never have used the existing health services or social support systems or may not have been diagnosed as having a DD. Second, we used only five years of administrative data to identify both DD cases and the mental health conditions of interest. A person with dementia or depression might not have had that particular diagnosis noted in their medical records within the five years of the study. As a result, this study would not have classified such a person as having dementia or depression. Having said that, it is important to note that MCHP researchers validated the dementia and depression operational case definitions based on the health administrative databases against other data sources (e.g., the Canadian Community Health Surveys) and found that they provide reliable prevalence estimates at the population level [55]. 

Third, although we matched the DD cases with non-DD controls based on age, sex, and place of residence for a more accurate and fair comparison, we selected our control group from the general population. This method of selecting the control group may have introduced some bias, leading to the higher risk ratios obtained in our study. We know that those accessing the health care system (using physicians or hospitals) may have higher rates of comorbidity; they may also receive more comprehensive medical examinations, which could increase their rates of diagnosed conditions—which, in fact, may have affected the rates estimated in this study. 

We want to acknowledge several other limitations of this study. We based our prevalence estimates on several health administrative databases, including data from physician claims. In remote areas of northern Manitoba, nurses (rather than physicians) deliver a high proportion of primary care; since administrative databases do not capture those services, even for mental health issues, this would skew our estimated rates. 

Also, as mentioned earlier in the Methods section, we excluded persons from the data sets who had DD and resided in a PCH or were listed with a Public Trustee postal code. Although we explained this decision, we acknowledge that it may have affected our results by excluding a disproportionate number of persons with DD in our original sample who had dementia. 

Despite these several acknowledged limitations, the present study stands as the first attempt to use provincial administrative databases to examine mental health issues for persons with DD at the population level. Accurate population-level information on the mental health status and conditions of persons with DD is needed for planning and providing services to meet the specific needs of this vulnerable population. The significant disparities this study found in rates of depression and dementia between persons with and without DD should alert health officials and authorities to the level of mental health needs of persons with DD. The existing policies regarding “community living” and “aging in place” encourage persons with disabilities and those with DD to access the mainstream health services, even for mental health problems. Studies which focused on access to specialized health services, including access to psychiatric services by persons with DD, remain rare, but these studies describe finding a number of barriers [24, 56]. To reduce disparities in mental health, those involved in planning and providing services to this population should focus on the barriers to accessing mental health services. Also, we must train health care professionals, including family physicians, to screen and assess mental health conditions and problems common among persons with DDs. Furthermore, we need more specialized training for medical staff, including physicians, to assist them with early detection and the provision of quality care to this population. 

Researchers recommend prevention as a strategy for reducing disparities in health, including mental health, between persons with and without DD (e.g., [57]). Preventive strategies should focus on modifiable risk factors, including promotion of physical activity, mental leisure activities, and social inclusion and participation—as well as providing meaningful job opportunities and adequate nutrition. Studies also highly recommend early diagnosis and detection of mental illnesses and management of symptoms and behaviours to minimize further complications [58]. Our research findings advocate for an increase in population-based epidemiological research studies targeting the prevalence of mental illnesses and disorders among individuals with DD. We need such research to better understand the extent and scope of mental illnesses in this population and how they vary by age, geography, sex, and diagnostic categories (e.g., mental retardation, ASDs, FASDs). We also suggest that future research consider access to health care, and in particular access to specialized care, as well as continuity of care. 

##  Disclosure

The results and conclusions are those of the authors, and no official endorsement by the Manitoba Centre for Health Policy, Manitoba Health, or other data providers is intended or should be inferred. 

## Figures and Tables

**Table 1:** Distribution of study subjects by age group.

Age group (years)	DD population		Matched non-DD		Total	
*n*	%	*n*	%	*n*	%

0–4	775	12.81	1,548	12.82	2,323	12.82
5–9	1,517	25.08	3,027	25.08	4,544	25.08
10–14	1,316	21.76	2,625	21.75	3,941	21.75
15–19	821	13.57	1,638	13.57	2,459	13.57
20–24	351	5.80	701	5.81	1,052	5.81
25–34	398	6.58	794	6.58	1,192	6.58
35–44	364	6.02	728	6.03	1,092	6.03
45–54	288	4.76	575	4.76	863	4.76
55–64	137	2.27	272	2.25	409	2.26
65+	81	1.34	161	1.33	242	1.34
Total	6,048	100.00	12,069	100.00	18,117	100.00

**Table 2:** Dementia prevalence (per 100 population), 2000/01–2004/05.

Study population and age groupings	Total *N*	Number with dementia	Prevalence (95% CI)
DD cohort			
20–54 Yrs	1,401	73	5.21 (4.05, 6.37)
55+ Yrs	218	30	13.76 (9.19, 18.33)

Matched comparison group (no DD)			
20–54 Yrs	2,798	39	1.39(0.96, 1.83)
55+ Yrs	433	15	3.46 (1.74, 5.19)

**Table 3:** Depression prevalence per 100 population, 2000/01–2004/05.

Study population and age group	Total *N*	Number with depression	Prevalence (95% CI)
DD cohort			
20–54 Yrs	1,401	595	42.47 (39.88, 45.06)
55+ Yrs	218	84	38.53 (32.07, 44.99)

Matched comparison group			
20–54 Yrs	2,798	559	19.98 (18.50, 21.46)
55+ Yrs	433	83	19.17 (15.46, 22.88)

**Table 4:** Age-specific depression and dementia RRs comparing those with a DD diagnosis to the matched cohort without a DD diagnosis.

	Risk Ratio	95% Confidence limits	
		Lower	Higher

Dementia			
20–54 Yrs	4.01*	2.72	5.92
55+ Yrs	4.80*	2.48	9.31

Depression			
20–54 Yrs	2.96*	2.59	3.39
55+ Yrs	2.65*	1.84	3.81

*P < 0.0001
